# Evaluation of sarcopenia, sarcopenic obesity, and phase angle in geriatric gastrointestinal cancer patients: before and after chemotherapy

**DOI:** 10.3906/sag-1808-114

**Published:** 2019-04-18

**Authors:** Derya HOPANCI BIÇAKLI, Rüksan ÇEHRELİ, Ahmet ÖZVEREN, Reci MESERİ, Rüçhan USLU, Mehmet UYAR, Bülent KARABULUT, Fehmi AKÇİÇEK

**Affiliations:** 1 Department of Medical Oncology, School of Medicine, Ege University, İzmir Turkey; 2 Department of Preventive Oncology, Institute of Oncology, 9 Eylül University, İzmir Turkey; 3 Department of Nutrition and Dietetics, School of Health, Ege University, İzmir Turkey; 4 Department of Anesthesia and Critical Care, School of Medicine, Ege University, İzmir Turkey; 5 Department of Geriatric, School of Medicine, Ege University, İzmir Turkey

**Keywords:** Geriatric oncology, sarcopenia, phase angle

## Abstract

**Background/aim:**

The purpose of this study was to determine sarcopenia, sarcopenic obesity and phase angle (PA) and the influence of chemotherapy (CT) on anthropometric measurements and and the PA in in geriatric patients with gastrointestinal (GI) cancer.

**Material and methods:**

The anthropometric measurements, calf circumference (CC), upper midarm circumference (UMAC), and hand grip strength (HGS), have been measured to understand muscle function of 153 patients (mean age of 70.5 ± 5.6 years, 28.8% female, 71.2% male). Sarcopenia and PA measurements have been evaluated by bioelectrical impedance analyses. The same evaluations were checked again after 1 cycle of CT (min: 4, max: 6 weeks).

**Results:**

Patient population consisted of colorectal (51,6%), gastric (26.8%), pancreas (11.8%), liver (7.2%), and biliary tract cancer (2%). UMAC (28.5 ± 4.4 before, 28.1 ± 4.9, P = 0.034 after CT), and HGS measurements (27.5 ± 8.6 before, 26.8 ± 8.8 after CT, P = 0.007) have significantly decreased after CT. CC measurement < 31 cm at first visit was seen in 13.1% of patients, but the ratio raised to 20.3% after CT (χ², P = 0.003). Severe sarcopenia was determined in 33% of all patients, and 30.0% of them have been considered as sarcopenic obese.

**Conclusion:**

Sarcopenia and sarcopenic obesity were prevalent in this group patients. The CT caused a decrease in muscle functions, UMAC, and CC. Patients should be followed up carefully for sarcopenia, sarcopenic obesity, and nutritional aspect and it would be proper to intervene before sarcopenia has not occurred yet.

## 1. Introduction

Sarcopenia causes one of the most noticeable changes in body composition together with aging (1), and it is a syndrome that may cause physical inadequacy, lowering of quality of life, increasing the risk of mortality, and it may appear with loss of strength as well as diffuse and progressive loss of muscle mass. When cancer is added to sarcopenia that appears together with aging, a complex process occurs. Severe loss of muscles is a condition accelerating tumor progression, influencing survival, and increasing frequency of toxicity due to chemotherapy (CT) in oncologic patients (2). Sarcopenic obesity, which is a subclass of sarcopenia, may cause limitation of functions and mobility in elderly (3) and increases mortality rates due to all reasons in women (4). There are approaches developed in diagnosing and evaluating sarcopenic patients (5). Bioelectrical impedance analysis (BIA) used in determination and evaluation of sarcopenia allows an easy and low-cost evaluation. Especially calf circumference (CC) of anthropometric methods gives an idea about regional and overall evaluation of skeletal muscle (6). The marker revealing the phase shifts during BIA measurement and giving the ratio of reactance to resistance angularly has been named as phase angle (PA). It has been put forward that PA is an indication of nutritional status together with body cellular mass (7). Studies show that bioelectrical PA is associated with morbidity and mortality in cancer patients (8,9,10). 

This study has been planned and conducted for the purpose of determining and evaluating sarcopenia, sarcopenic obesity, and PA in geriatric patients with gastrointestinal (GI) cancers. Secondary aim of the study is to determine the influence of CT and malnutrition on body composition (body fat percent and muscle amount) and PA.

## 2. Materials and methods

In this prospective and descriptive study, all geriatric patients (≥ 65 years old) with a diagnosis of GI cancer, who receive CT and do not have hearing problem, were evaluated between October 2015 and January 2016 in Ege University Medical Faculty Hospital, Medical Oncology outpatient clinic in İzmir, Turkey. The exclusion criteria were as follows: patients <65 years, pacemakers, significant ascites, edema, inability to stand up, and hearing and perception problems. Weakness status of these patients was published previously (11). This paper produced seconder analysis from original data of the same patients. This study received approval from the University Scientific Research Ethics Committee and permission from the Department of Medical Oncology. Anthropometric measurements such as CC and upper midarm circumference (UMAC) were taken to estimate skeletal muscle mass (6), and nutritional status was determined by mini nutritional evaluation (MNA) survey (12) on the first visit and on the visit after receiving CT. The time between the two visits was set to be 4 min to maximum 6 weeks. Nutritional status was classified as good (>24 points), having risk of malnutrition (17–24 points), and in malnutrition (<17 points). Body composition analysis and PA values of patients were carried out by Tanita MC 780 BIA device. Skeletal muscle mass (kg) was calculated with the formula validated previously (13). Values normalized for height (kg/m2) were classified as severe sarcopenia (≤5.75 for females and ≤8.50 for males), moderate sarcopenia (5.76–6.75 for females and 8.51–10.75 for males), and normal (≥6.76 for females and ≥10.76 for males) (14,15,16). Skeletal muscle index (SMI) is calculated by the formula of muscle mass/height2 of patients. UMAC was measured by tape measure from the midpoint between acromial and olecranon protrusions in standing upright position while arm is twisted 90° from elbow. UMAC measurements of <21.1 in males and <19.2 in females were considered as signs of sarcopenia (17). CC measurements were made by tape measure from the widest part of legs by pressing feet onto a hard and plain ground. Thirty-one cm was accepted as threshold value for calf circumference (18). Threshold values of < 30 kg for males and <20 kg for females for hand grip strength were used to assess muscle function (Takei grip strength dynamometer, Japan). 

Patients with a body mass index (BMI) of 25 to 29 were accepted as mild obese, whereas the ones with a BMI of > 30 as obese. If a patient was both mild obese/obese and sarcopenic, he/she was defined as a “sarcopenic obese”.

Continuous variables were compared by t test in dependent groups (by Wilcoxon signed-rank test parametric conditions were not met). McNemar chi-square test was used for classified data. Ability of CC for predicting sarcopenia compared to sarcopenia determined by BIA was evaluated with receiver operating characteristic (ROC) curve. Sex-specific cut points were determined according to Youden index. The point having the highest value of Youden index (J = Sensitivity + Selectivity – 1) was determined as the cut point. P < 0.05 was accepted as statistically significant. 

## 3. Results

A total of 153 patients (mean age 70.5 ± 5.6 years, 29% female) participated in this study. The most common cancer diagnoses were consisted of colorectal (51.6%), stomach (26.8%), and pancreas (11.8%) cancers. Stage 4 cancer patients were 121 (79.1%), whereas 26 (17%) and 6 (3.9%) had stage 3 and stage 2 cancer, respectively. One-hundred twenty-two patients (79.5%) were already receiving CT and 31 patients (20.3%) had recently initiated CT. Radiotherapy and surgery were applied as a part of the cancer treatment for 37 (24.2%) and 92 patients (60%), respectively. 

The patients’ sarcopenia frequencies are shown in Table 1. Severe and moderate sarcopenia were found in 39 (33%) and 88 patients (58%), respectively. Moderate or severe sarcopenia was found in 80.7% of patients having initial CT and in 83.6% of patients already on CT, but difference is not statistically significant (P = 0.500). 

**Table 1:** Frequency and classification of sarcopenia in patients.

Sarcopenia Status	Female	Male
	n %	n %
Severe Sarcopenia	6 13.6	33 30.3
Moderate Sarcopenia	17 38.6	71 65.1
Normal	21 47.7	5 4.6
Total	44 100.0	109 100.0

Sixty-four patients (41.8%) were mild obese or obese. Of these patients, 71.9% (n = 46) were also sarcopenic. In other words, 30.0% of all patients were evaluated as sarcopenic obese. 

Anthropometric measurements are shown in Table 2. UMAC and CC measurements of patients decreased significantly after CT. CC below 31 cm was measured in 13.1% and 20.3% of the patients on the first visit and on the visit after receiving CT, respectively (McNemar χ², P = 0.003). While 54.5% of the female patients had inadequate HGS as an indicator of muscle function at the first visit, this ratio was 56.8% after CT. A total of 50.5% of the male patients had inadequate HGS at the first visit, but this ratio was 55.0% after receiving CT. When classified according to the mentioned cut points, the difference was not found to be statistically significant (P = 0.327). However, when HGS values on average were evaluated without a cutting point, first measurement was 27.5 ± 8.6 kg, whereas second measurement was found to be 26.8 ± 8.8 kg indicating a statistically significant decrease in muscle strength (P = 0.007). Body fat percentages of patients with and without malnutrition according to MNA at the baseline and after receiving CT are shown in Table 3. The body fat percentage of the male patients with and without malnutrition was significantly decreased after CT and the fat percentage of female patients without malnutrition increased significantly after CT ( P = 0.003). PA values of the patients at first visit and after receiving CT are demonstrated in Table 4. Change in PA values according to sex was not found to be statistically significant.

**Table 2:** Anthropometric measurements of patients at first visit and after receiving CT.

Anthropometric Measurements	First Measurement	Measurement after CT	P*
	Ort ± SD	Ort ± SD	
UMAC	28.5 ± 4.4	28.1 ± 4.9	0.034
CC	34.8 ± 4.2	33.9 ± 4.2	< 0.001

UMAC: Upper midarm circumference, CC: Calf circumference. * t test in dependent groups.

**Table 3:** Fat percent values of patients at first visit and after receiving CT.

	Fat percentages of patients without malnutrition			Fat percentages of patients having malnutrition		
	First visit	After CT		First visit	After CT	
	Mean ± SD	Mean ± SD	p	Mean ± SD	Mean ± SD	P
Male	20.74 ± 10.20	16.78 ± 8.05	0.002	13.70 ± 9.66	13.02 ± 9.41	0.001
Female	22.60 ± 9.44	28.86 ± 8.45	0.003	19.12 ± 8.08	18.56 ± 8.13	0.128

* t test in dependent group.

**Table 4:** PA values of patients at first visit and after receiving CT.

	PA Values		
Sex	First visitMean ± SD	After CT Mean ± SD	P
Male	4.96±0.92	4.94±0.93	0.834
Female	4.73±0.72	4.60±0.70	0.309

* t test in dependent group.

Influence of malnutrition on skeletal muscle index and PA are shown in Table 5. According to this table, the patients with malnutrition had lower PA and SMI values than the patients with normal nutritional level.

**Table 5:** Effect of malnutrition on SMI (kg/m2) and PA.

Malnutrition				
		AbsentMean ± SD	PresentMean ± SD	P*
First visit	PA	5.09 ± 0.78	4.56 ± 0.91	<0.001
	SMI	8.82 ± 1.41	7.74 ± 1.45	<0.001
After CT	PA	5.17 ± 0.75	4.47 ± 0.87	<0.001
	SMI	8.87 ± 1.50	8.04 ± 1.65	0.001

* t test in independent groups.

Average PA (P < 0.001) and SMI values (P < 0.001) in patients with malnutrition determined on the first measurement were found to be significantly low. 

### 3.1. Association of sarcopenia determined by BIA with CC 

While there was a strong (r = 0.598) and significant (P < 0.001) correlation in positive direction of SMI (amount of muscle mass/height2) and CC in males in measurements after CT, a very strong (r = 0.759) and significant (P < 0.001) correlation in positive direction was determined in female patients. Ability of CC measured after receiving CT for predicting the existence of sarcopenia obtained by BIA was examined separately in males and females, and ROCs are presented in the Figure.

**Figure:**
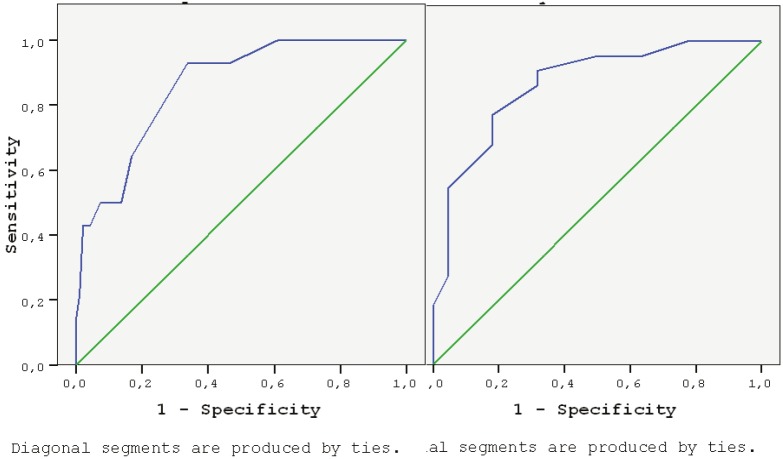
Ability of CC measured after receiving CT for estimating the presence of sarcopenia obtained by BIA for male and female patients.

## 4. Discussion

Our study evaluated sarcopenia, prevalence of sarcopenic obesity, PA, and their interrelations with CT in geriatric patients with GI cancer, who represent a risky group for sarcopenia, and sarcopenia prevalence was found to be high in geriatric patients with GI cancer and patients who were receiving CT for a longer time were under greater risk. In addition, prevalence of “sarcopenic obesity” was assessed, which is generally a neglected condition. This is the first geriatric oncology study putting light on this aspect.

Previous prevalence studies have used various methods and diagnostic criteria, including dual energy X ray absorptiometry (DXA), BIA, and anthropometric measurements and indicated sarcopenia prevalence between 0.0%-85.4% in males and 0.1%–33.6% in females. The prevalence in studies performed by using DXA was found to be 0.0%–56.7% in males and 0.1%–33.9% in females, 6.2% and 85.4% in males and 2.8% and 23.6% in females in studies performed by using BIA (19). Our study found that 33% of the patients had severe sarcopenia. 

There are several factors that may affect the significant reduction in the frequency of males with sarcopenia. The increasing body water especially extracellular water in cancer may cause exaggerating or ignoring body weight, muscle mass, and lean body mass (19). Body water also may have an impact on the prevalence of sarcopenia between females and males. Furthermore, urinary parameters in advanced cancers may change due to mostly impaired renal functions and diuretics (20). One of the other factors affecting the status of sarcopenia is the level of patients’ physical activity. The prevalence of physical activity was reported in older men and women, and the level of physical activity of men was higher than women (21). In our study, physical activity, which is one of the parameters that can explain the decrease in male patients with sarcopenia after chemotherapy, has not been evaluated.

 CC measurements are considered to have a positive relation with muscle mass and a negative relation with the status of disability with an easy, cheap, and valuable application. Changes in fat stores in elderly, decrease of skin elasticity, and continuous change of measuring people may cause measurement errors. Measurements made by professionals trained on the subject and, if possible, measurements made by the same people decrease the possibility of errors (2). In our study, first and second measurements of all patients were made by the same researcher, thus eliminating measurement differences. This is considered as a strong point of this study. 

In the study of Landi et al. on 357 geriatric individuals, in which relation of midarm muscle area was calculated by a formulation with UMAC and skin fold and mortality was evaluated, it has been found that physical performance and survival time have a significant relation with upper midarm area with higher values indicating increased survival (22). In our study, a significant decrease was found in anthropometric measurements between the first visit and after receiving CT in anthropometric measurements (CC, UMAC) and hand grip strength that was used for determining muscle strength. 

The European Working Group of Sarcopenia on Older Population (EWGSOP) has published a sarcopenia consensus report in 2010, indicating that there should also be a decrease of muscle functions together with the reduction of muscle mass for the diagnosis of sarcopenia (2). Even in European consensus report, subjects that which parameters would be used such as fat-free mass, muscle mass, total muscle mass of arms and legs in formulas used were based on BIA, via what megahertz (MHz) resistance and reactance would be calculated, and therefore the subject of PA determination has not been definitely recommended.

In our study, it was determined that approximately a quarter of oncology patients did not have sarcopenia and the remaining had moderate or severe sarcopenia. In a review examining 53 studies including 9138 patients, in which changes in body composition and its results on oncology have been investigated, it has been indicated that loss of fat-free mass and sarcopenia negatively influence the outcome of cancer treatment and increase CT toxicity and postoperative complications (23). Sarcopenia is seen in 20%–70% of cancer patients, whereas 40-60% of patients are overweight or obese. This makes the determining and recognizing muscle loss more difficult (24). Prevalence of sarcopenic obesity has been found to be 18.1% in females and 42.9% in males (4). In a study trying to put forward the prevalence of sarcopenic obesity in patients with cancer, functional capacity of sarcopenic obese patients has been found to be lower than nonsarcopenic obese ones, and it has also been indicated that this condition is an independent predictor for survival (25). In our study, it was found that 30.0% of all patients were sarcopenic obese. Sarcopenic obese patients, who are neglected in malnutrition approach that mostly evaluates only weakness and who are mostly not recognized, should be carefully screened and followed up.

Skeletal muscle is the largest organ in human body. Although cachexia characterized by muscle loss is known for a long time in oncology, relation of muscle mass with systemic inflammation, inadequate nutrition, and disease prognosis has only been demonstrated in recent years. Sarcopenia, independent of the cancer stage, is an important prognostic factor (26). In our study, SMI values of patients on the first visit showed difference in patients with and without malnutrition. 

In our study, prevalence moderate or severe sarcopenia was found in 80.7% of patients having CT for the first time and in 83.6% of patients having CT for a long time. This lack of significant difference may be associated with a short follow-up time. Studies with longer follow-up are needed in order to understand the effects of CT. 

Body composition analysis by BIA shows fluid balance of the body and its status of cellular health in a noninvasive and fast way. While reactance measured by BIA is associated with protective cellular membrane, resistance is associated with total body tissue fluid. While high PA score reflects good function of cellular membrane, low PA is closely associated with cellular apoptosis and a decrease in content of cellular matrix (27). In our study, it is estimated that a short follow up period may have influenced the fact that the PA average didn’t change statistically significantly between the first visit and after CT. It has been found in the study of Gupta et al. that influence of PA is significant and that median survival has been found as 40.4 months in those with PA of >5.57, while median survival has been 8.6 in those with PA of ≤5.57 (28). In another study, it has been indicated that PA is an independent marker in patients with breast cancer and that nutritional interventions targeting improvement of PA may increase survival in patients with breast cancer (9). In our study, PA value of patients without malnutrition was found to be higher compared to patients with malnutrition both at first visit and after CT. In our study we found a significant increase in body fat percentage in female patients without malnutrition after CT. We didn’t record and evaluate hyperthyroidism, diabetes mellitus such as diseases that can affect the percentage of body fat, and the use of drugs that may cause fat accumulation. This situation prevented the discussion of the fat percentage of the patients. In addition, the short duration of our study was the limitation of our study.

There is a positive correlation between CC and body muscle content, and CC below 31 cm has been associated with disability in elderly (18). In our study, when the correlation between CC and BIA was assessed, CC appeared to be an anthropometric measurement that could be measured easily and could predict sarcopenia in daily practice. 

In conclusion, in the light of the data obtained in this study, it is determined that sarcopenia and sarcopenic obesity are prevalent in patients with geriatric GI cancer and even a single period of treatment influences body composition negatively. By taking this result into account, this group of patients should be followed up closely for the development of sarcopenia, sarcopenic obesity, and cachexia, which are intertwined and difficult to distinguish. Further investigations for possible mechanism of sarcopenia in geriatric cancers are required. It may be possible to prevent the sarcopenia and advance treatments for very prevalent sarcopenia via better recognizing underlying mechanisms. Today, while all variables that may influence prognosis in cancer patients are investigated in detail, investigating the influence of sarcopenia into prognosis may add a new perspective to oncological treatments. The investigation for preventing malnutrition and sarcopenia should be managed by multidisciplinary teams including health professionals such as doctors, dietitians, nurses, pharmacists, physiotherapists, and psychologists, where interdisciplinary exchange of knowledge is performed.
